# Regulation of XPC deubiquitination by USP11 in repair of UV-induced DNA damage

**DOI:** 10.18632/oncotarget.22105

**Published:** 2017-10-29

**Authors:** Palak Shah, Lei Qiang, Seungwon Yang, Keyoumars Soltani, Yu-Ying He

**Affiliations:** ^1^ Department of Medicine, Section of Dermatology, University of Chicago, Chicago, IL, USA; ^2^ Committee on Molecular Pathogenesis and Molecular Medicine, University of Chicago, Chicago, IL, USA

**Keywords:** USP11, XPC, UVB, nucleotide excision repair, skin cancer

## Abstract

Nucleotide excision repair (NER) is the most versatile DNA repair pathway for removing DNA damage caused by UV radiation and many environmental carcinogens. NER is essential for suppressing tumorigenesis in the skin, lungs and brain. Although the core NER proteins have been identified and characterized, molecular regulation of NER remains poorly understood. Here we show that ubiquitin-specific peptidase 11 (USP11) positively regulates NER by deubiquitinating xeroderma pigmentosum complementation group C (XPC) and promoting its retention at the DNA damage sites. In addition, UV irradiation induces both USP11 recruitment to the chromatin and USP11 interaction with XPC in an XPC-ubiquitination-dependent manner. Furthermore, we found that USP11 is down-regulated in chronically UV-exposed mouse skin and in skin tumors from mice and humans. Our findings indicate that USP11 plays an important role in maintaining NER capacity, and suggest that USP11 acts as a tumor suppressor *via* its role in DNA repair.

## INTRODUCTION

Nucleotide excision repair (NER) is the most versatile DNA repair system for removing various forms of bulky DNA damage induced by environmental carcinogens, including solar ultraviolet B (UVB) radiation and air pollutants [1-3]. NER has two subtypes based on the location of the damage in DNA: global genome nucleotide excision repair (GG-NER), which removes damage from the entire genome, and transcription coupled nucleotide excision repair (TC-NER), which removes damage from actively transcribed regions of the genome [4, 5]. Defective GG-NER in humans leads to the Xeroderma pigmentosum (XP) syndrome, characterized by an increased risk of carcinogenesis in various organs including the skin, lungs and brain [4-7]. The risk is especially increased significantly at a very young age for non-melanoma and melanoma skin cancers, the most common cancer in the United States [8-10]. Of the xeroderma pigmentosum complementation group A-G (XPA-XPG) factors identified in the GG-NER process, XPC plays a vital role in the initial DNA damage recognition step in GG-NER [4-6, 11-13], and in preventing carcinogenesis in various organs, especially skin carcinogenesis [14-18].

XPC is regulated at various levels including genetic, transcriptional, post-translational, and by immunosuppression [19]. After UV exposure, XPC is polyubiquitinated by the UV-DDB E3 ligase complex, consisting of DDB1 (DNA damage-binding protein 1), DDB2, CUL4A (Cullin-family E3-ligase adaptor protein) and ROC1 (E3-ligase RING domain) [20-24]. Ubiquitination of XPC by the UV-DDB complex enhances XPC binding to the DNA damage site and is essential for its DNA damage recognition function in NER [20, 21, 24]. Subsequently XPC is sumoylated and then undergoes a second ubiquitination event by RING finger protein 111 (RNF111), which mediates XPC release from the damage site, and also promotes efficient NER [23-27]. Since ubiquitination of XPC does not promote XPC degradation, it must probably be deubiquitinated and recycled [21]. Even though the biochemical function of XPC in the NER process has been extensively studied, regulation of XPC by deubiquitination is largely unknown. Only recently, USP7 was identified as a deubiquitinase for XPC, which promoted the NER process [28]. Identifying novel regulators of XPC deubiquitination could provide more drug-susceptible targets than XPC to modulate XPC activity in the NER process and prevent skin cancer.

Ubiquitin specific peptidase 11 (USP11) is a member of the ubiquitin-specific proteases (USPs) family of deubiquitinase enzymes [29]. USP11 participates in various signaling pathways and biological processes such as TGFβ signaling, pro-inflammatory signaling, viral replication, and NF-κB signaling, by regulating deubiquitination and protein stability of various targets such as TβRII, ALK5, LPA1, NP protein, and IκBα [30-34]. Additionally, USP11 has emerged to positively regulate DNA double-strand break (DSB) repair by regulating PALB2 deubiquitination, by modifying recruitment of RAD51 and 53BP1 to the DNA damage site dependent on its catalytic activity, and by interacting with BRCA2 independent of its catalytic activity [35-38]. Proteomic analysis by Havugimana and colleagues predicted that USP11 and XPC interact as part of a protein complex [39]. However, the regulatory and functional role of USP11 in NER is unknown. The objective of this study was to determine the role of USP11 in the NER pathway. We further determined whether the mechanism of USP11 activity was *via* regulating deubiquitination of XPC, as well as USP11’s role in skin cancer.

## RESULTS

### USP11 promotes UVB-induced DNA damage repair

**Figure 1:**
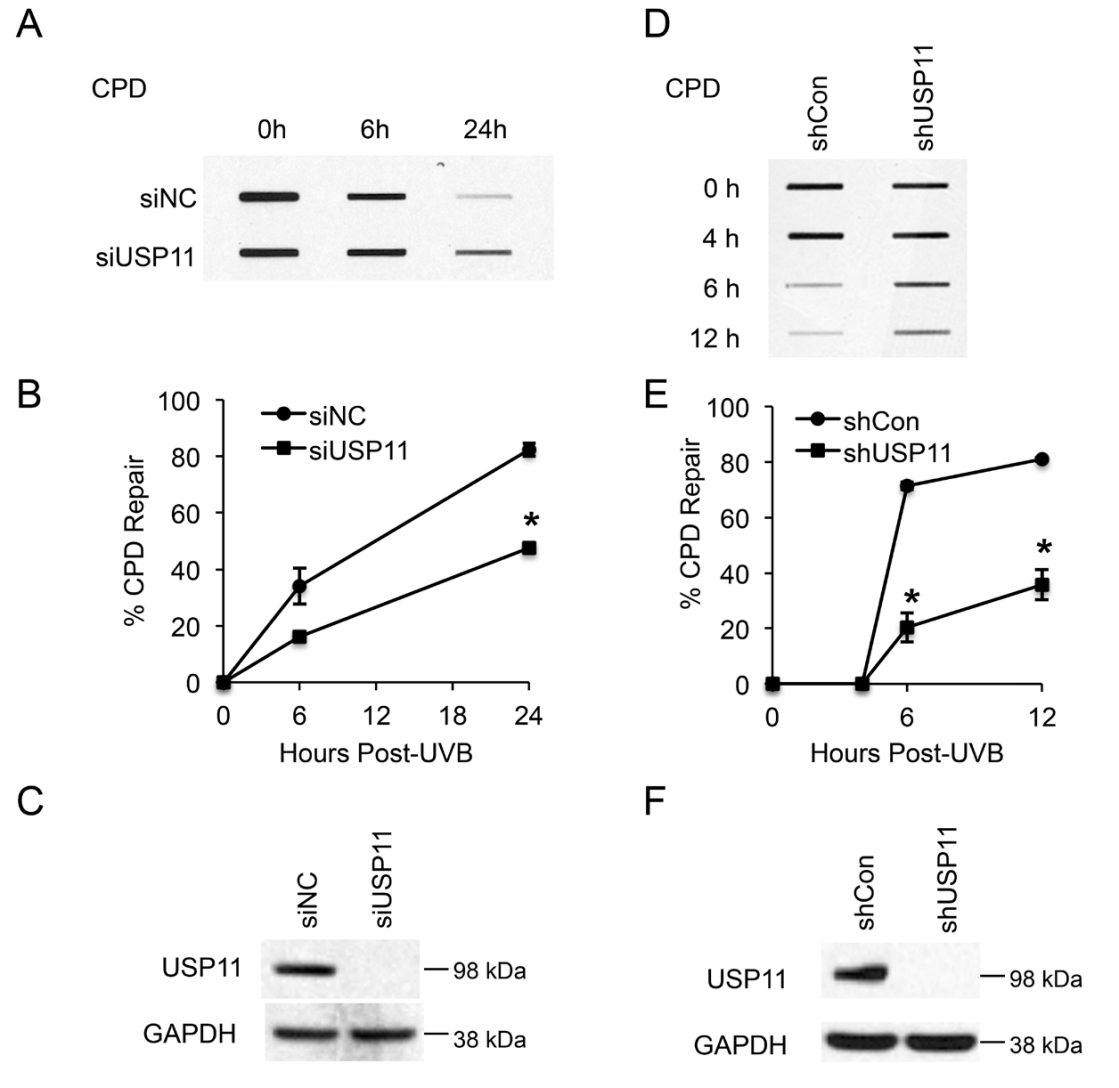
USP11 promotes UV-induced DNA damage repair (**A**, **D**) Slot blot analysis of the levels of CPD at indicated times post-UVB (20 mJ/cm^2^) in HaCaT cells transfected with siRNA targeting USP11 (siUSP11) or non-targeting control siRNA (siNC) (A), and HaCaT cells stably infected with a lentiviral vector expressing control shRNA (shCon) or shRNA targeting USP11 (shUSP11) (D). (**B, E**) Quantification of percentage (%) of CPD repair (B) from (A), and (E) from (D) (mean ± S.E., n=3). *, *P* < 0.05, compared with siNC and shCon groups respectively, Student’s *t*-test. (**C, F**) Immunoblot analysis of USP11 and GAPDH in HaCaT cells transfected with siUSP11 or siNC (C), and stably infected with a lentiviral vector expressing shCon or shUSP11 (F).

To determine whether USP11 affects repair of UVB-induced DNA damage, we measured the difference in UV-induced DNA damage repair between control and USP11-inhibited cells. We focused on the repair of cyclobutane pyrimidine dimers (CPD), since CPD is the main photoproduct of UV-induced DNA damage in humans, and unrepaired CPD damage leads to skin cancer [40]. In HaCaT cells, both siRNA- and shRNA-mediated USP11 knockdown significantly inhibited CPD repair (Figure 1A-1F, *P* < 0.05, Student’s *t*-test). The experimental conditions and low dose for UV radiation (20 mJ/cm^2^) were chosen to avoid significant changes in cell proliferation and apoptosis after UV exposure, which could affect DNA damage measurements (data not shown). Our results indicate that USP11 positively regulates repair of UV-induced CPD DNA damage, and suggests a tumor suppressive function of USP11 in skin cancer.

### USP11 deubiquitinates XPC at the chromatin following UVB damage

**Figure 2:**
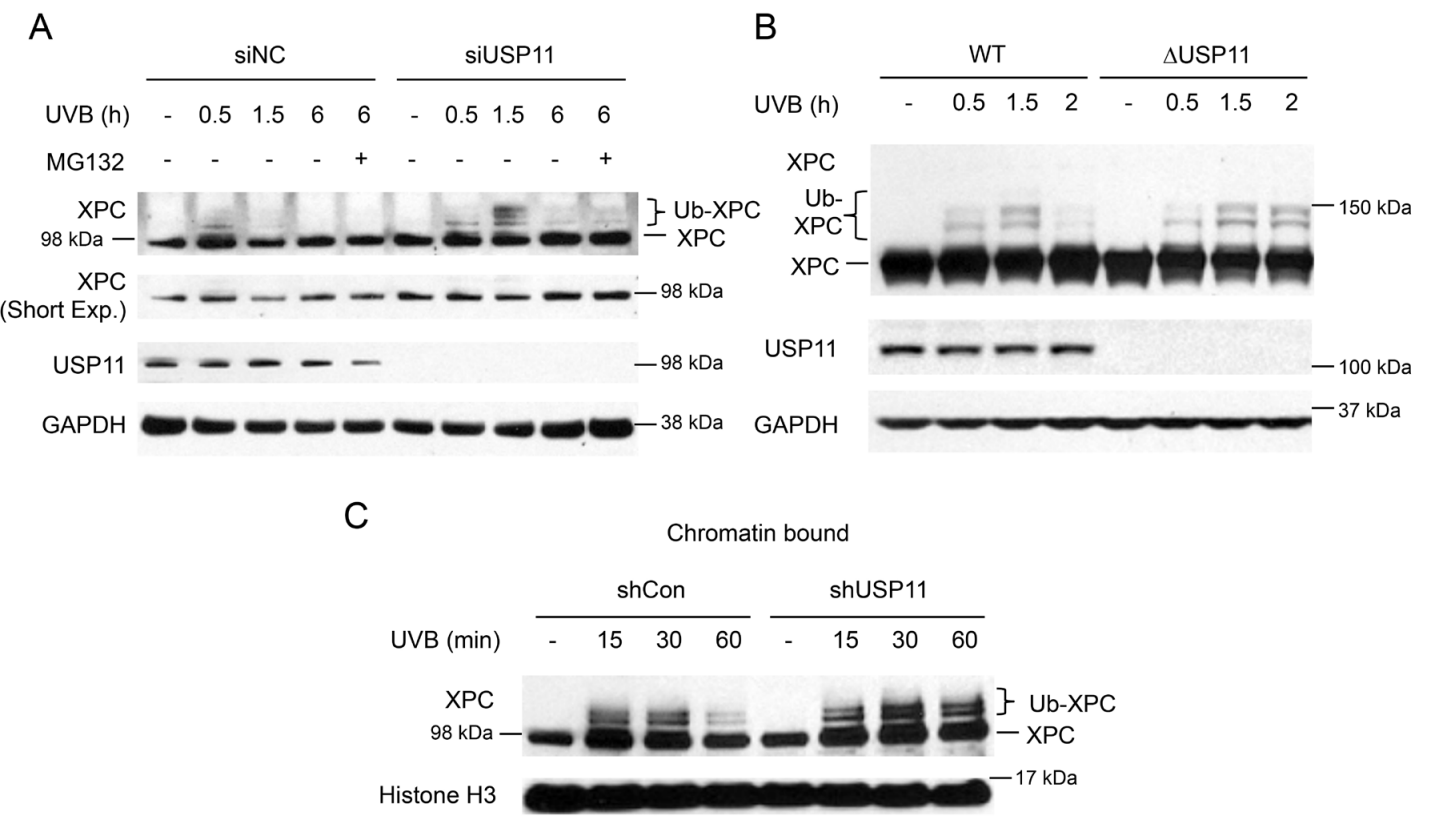
USP11 deubiquitinates XPC at the chromatin following UVB damage (**A, B**) Immunoblot analysis of XPC, USP11, and GAPDH in HaCaT cells transfected with siUSP11 or siNC and treated with or without MG132 (10 µM) 1 h prior to UVB exposure (20 mJ/cm^2^) (A), 293T WT and ΔUSP11 cells (B) at the indicated times post-UVB (20 mJ/cm^2^). (**C**) Immunoblot analysis of XPC and histone H3 using chromatin-bound protein fractions from HaCaT cells stably infected with a lentiviral vector expressing shCon or shUSP11 at the indicated times post-sham or -UVB (20 mJ/cm^2^) irradiation.

To elucidate the mechanism by which the deubiquitinase USP11 affects UVB-induced DNA damage repair, we determined whether USP11 regulates deubiquitination of XPC after UVB exposure, since ubiquitination of XPC is important for its efficient function in the NER process [21, 26]. Using biochemical and biological approaches, Sugasawa and colleagues have demonstrated that UV induces XPC ubiquitination through the UV-DDB complex, and that XPC immunoblot analysis detects several bands for ubiquitinated XPC (migrated slower than non-ubiquitinated XPC), in addition to non-ubiquitinated XPC [21]. Thus we used this immunoblot method to detect XPC ubiquitination. In HaCaT keratinocytes, knockdown of USP11 by siRNA increased ubiquitinated XPC levels at 1.5 h post-UVB as compared to control siRNA, while it did not affect the levels of XPC ubiquitination at 0.5 h post-UVB (Figure 2A). This indicates that USP11 is important for XPC deubiquitination after UV exposure. Since at 6 h the XPC ubiquitination levels decreased to a similar level in both the siUSP11 and control groups, XPC was eventually deubiquitinated in the siUSP11 group, possibly by a mechanism independent of USP11. Previous studies have shown that XPC is degraded after UV damage [25]. With MG132 proteasome inhibitor treatment, the siUSP11 group did not show difference in XPC levels compared to control, indicating that USP11 does not affect degradation of XPC after UV damage. Similarly, after UV damage in 293T ΔUSP11 cells, USP11 deficiency increased XPC ubiquitination levels only at a later time point (2 h) after UV damage compared to WT cells, not at earlier time points (0.5 h and 1.5 h) (Figure 2B). Previous studies indicate that XPC is recruited to the DNA damage site and is ubiquitinated to promote its binding to the damage site [21]. To confirm that changes in XPC ubiquitination by USP11 occur at the chromatin, we examined changes in XPC ubiquitination by USP11 in the chromatin-bound XPC protein fraction. In the chromatin-bound protein fraction, USP11 knockdown increased ubiquitination of XPC at a later time point (60 min), but not earlier ones, as compared with control (shCon) cells (Figure 2C). Our findings indicate that USP11 mediates XPC deubiquitination at the chromatin following UVB damage.

### Catalytic activity of USP11 is essential to regulation of XPC deubiquitination and NER after UVB exposure

**Figure 3:**
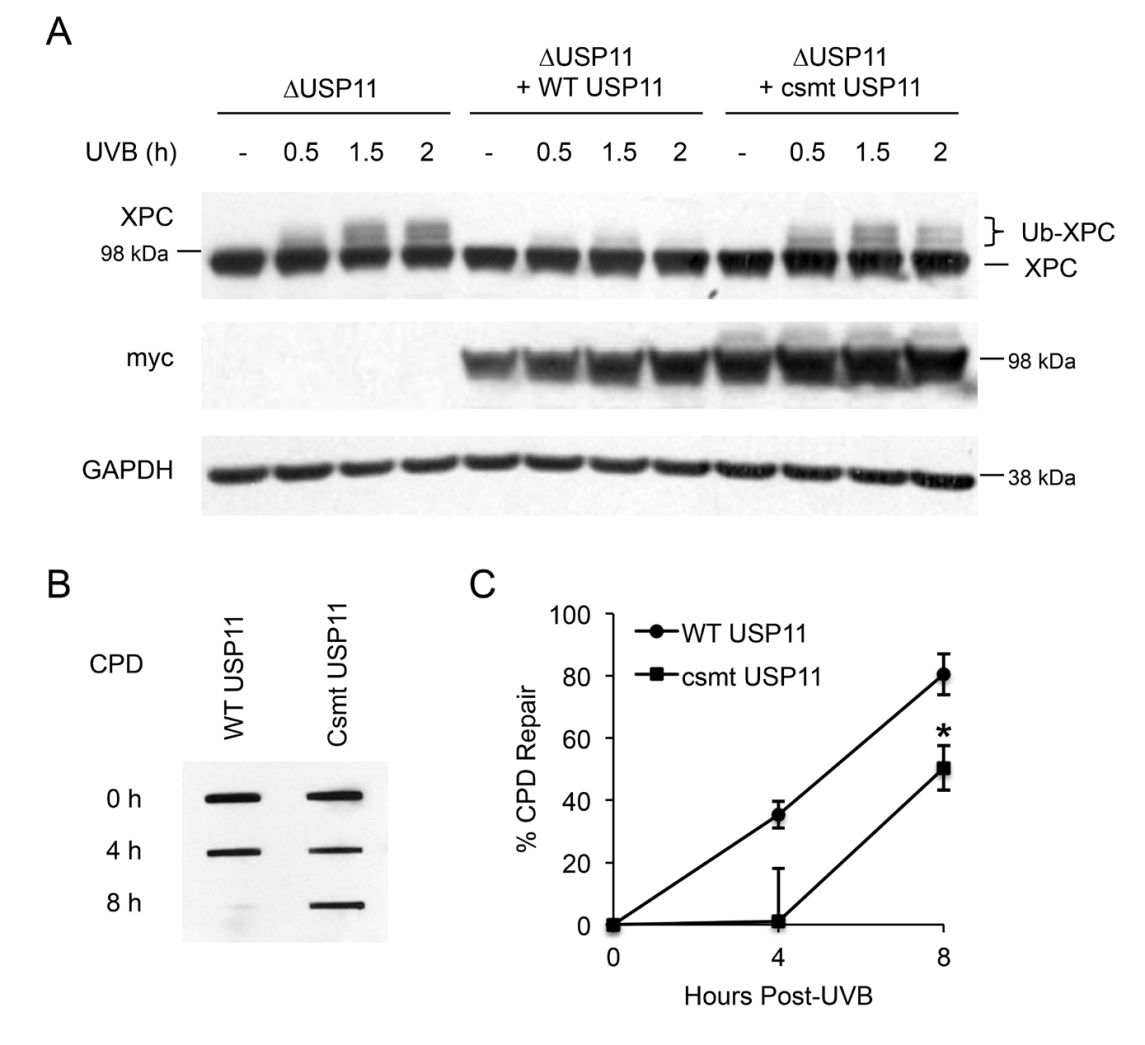
Catalytic activity of USP11 is essential to regulating XPC deubiquitination and NER after UVB exposure (**A**) Immunoblot analysis of XPC, myc, and GAPDH in 293T ΔUSP11 cells transfected with or without myc- tagged wild-type (WT) and catalytic mutant USP11 plasmids (csmt USP11) at the indicated times post-UVB (20 mJ/cm^2^) or -sham irradiation. (**B**) Slot blot analysis of the CPD levels at indicated times after UVB (5 mJ/cm^2^) in 293T ΔUSP11 cells transfected with WT USP11 or csmt inactive mutant USP11 plasmids. (**C**) Quantification of percentage (%) of CPD repair from (B) (mean ± S.E., n=3). *, *P* < 0.05, compared with WT USP11 group, Student’s *t*-test.

To determine whether the deubiquitinase activity of USP11 is necessary for USP11-mediated deubiquitination of XPC, we assessed the difference in XPC ubiquitination levels after UV damage between wild-type USP11- and C275/283S mutant (csmt) USP11-added 293T cells with USP11 genetic deletion (ΔUSP11). The C275/283S mutant USP11 is a catalytically inactive mutant of USP11. Expression of wild-type USP11 decreased XPC ubiquitination levels, whereas expression of csmt USP11 had little effect (Figure 3A). This indicates that the catalytic activity of USP11 is essential for USP11-mediated deubiquitination of XPC after UVB. Additionally, csmt USP11 expression in 293T ΔUSP11 cells showed decreased CPD repair after UVB irradiation as compared with WT USP11-expressing cells (Figure 3B and 3C), indicating that the catalytic activity of USP11 is vital for its effect on CPD repair. These results indicate that the catalytic activity of USP11 is necessary to mediate XPC deubiquitination and to promote UV-induced DNA damage repair.

### USP11 knockdown leads to premature dissociation of XPC from DNA damage sites by VCP/p97

**Figure 4:**
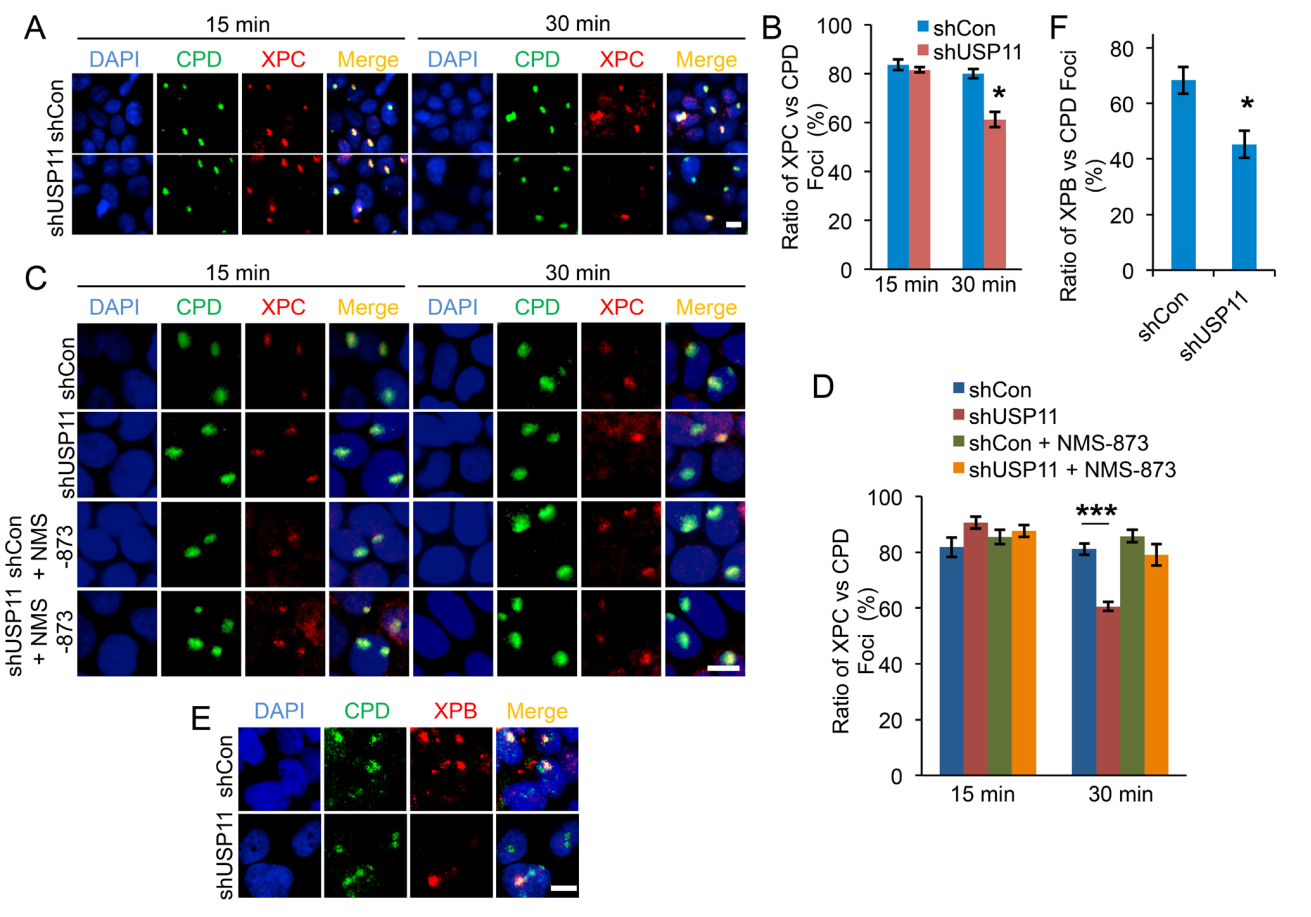
USP11 knockdown leads to premature dissociation of XPC from the DNA damage sites by VCP/p97 (**A, C**) Immunofluorescence assay of the colocalization of XPC with sub nuclear CPD in HaCaT cells stably infected with a lentiviral vector expressing shCon or shUSP11 at 15 min, 30 min post-UV (10 mJ/cm^2^) through a 5 μm micropore filter (A) or cells pretreated with NMS-873 (10µM) or vehicle for 1 hour prior to UV exposure (C). Scale bar, 10 μm. (**B, D**) The ratio of XPC to CPD foci (B) from (A), and (D) from (C) were calculated by analyzing 100 foci for merged fluorescent signals of XPC and CPD foci (n= 100, error bar: S.E.). *, *P* < 0.05, compared with shCon group, Student’s *t*-test. The results were obtained from three independent experiments. (**E**) Immunofluorescence assay of the colocalization of XPB with sub nuclear CPD in HaCaT cells stably infected with a lentiviral vector expressing shCon or shUSP11 at 30 min post-UV (10 mJ/cm^2^) through a 5 μm micropore filter. Scale bar, 10 μm. (**F**) The ratio of XPB to CPD foci from (E) was calculated by analyzing 100 foci for merged fluorescent signals of XPB and CPD foci (n= 100, error bar: S.E.). *, *P* < 0.05, compared with shCon group, Student’s *t*-test. The results were obtained from three independent experiments.

Next we determined whether USP11 affected XPC localization to the DNA damage site. We used a local UV radiation method, in which cells were exposed to UV radiation through a micropore filter leading to the formation of sub-nuclear localized DNA damage foci, and we evaluated colocalization of XPC to the CPD DNA damage foci. In both shCon and shUSP11 HaCaT cells, similar amounts of XPC colocalized with CPD damage foci at 15 min post-UV irradiation (Figure 4A and 4B). However, at a later time point (30 min) after UV exposure, shUSP11 cells showed significantly reduced colocalization of XPC with CPD foci as compared with shCon cells. These results indicate that USP11 knockdown mediates premature dissociation of XPC from the DNA damage site, while it does not affect XPC recruitment to the damage site. To determine the mechanism by which USP11 affects XPC dissociation from the damage site, we asked whether VCP/p97 might play a role, since VCP/p97 had been found to mediate XPC removal from DNA damage sites [41]. We determined the difference in XPC localization to CPD damage foci between HaCaT shCon and shUSP11 cells with or without a potent and specific inhibitor for VCP, NMS-873 [42]. We found that NMS-873 pretreatment inhibited the effect of USP11 deficiency on premature dissociation of XPC from the DNA damage site (Figure 4C and 4D). These findings demonstrate that USP11 promotes proper retention of XPC at the DNA damage site by preventing VCP/p97-dependent XPC removal. To determine whether the effect of USP11 on XPC retention affects the downstream NER pathway, we assessed the impact of USP11 on recruitment of XPB to the damage site. shUSP11 HaCaT cells showed significantly reduced colocalization of XPB with CPD foci as compared with shCon cells at 30 min post-UV irradiation (Figure 4E and 4F). These results indicate that USP11 knockdown decreases recruitment of XPB to the DNA damage site.

### UVB induces USP11 recruitment to the chromatin

**Figure 5:**
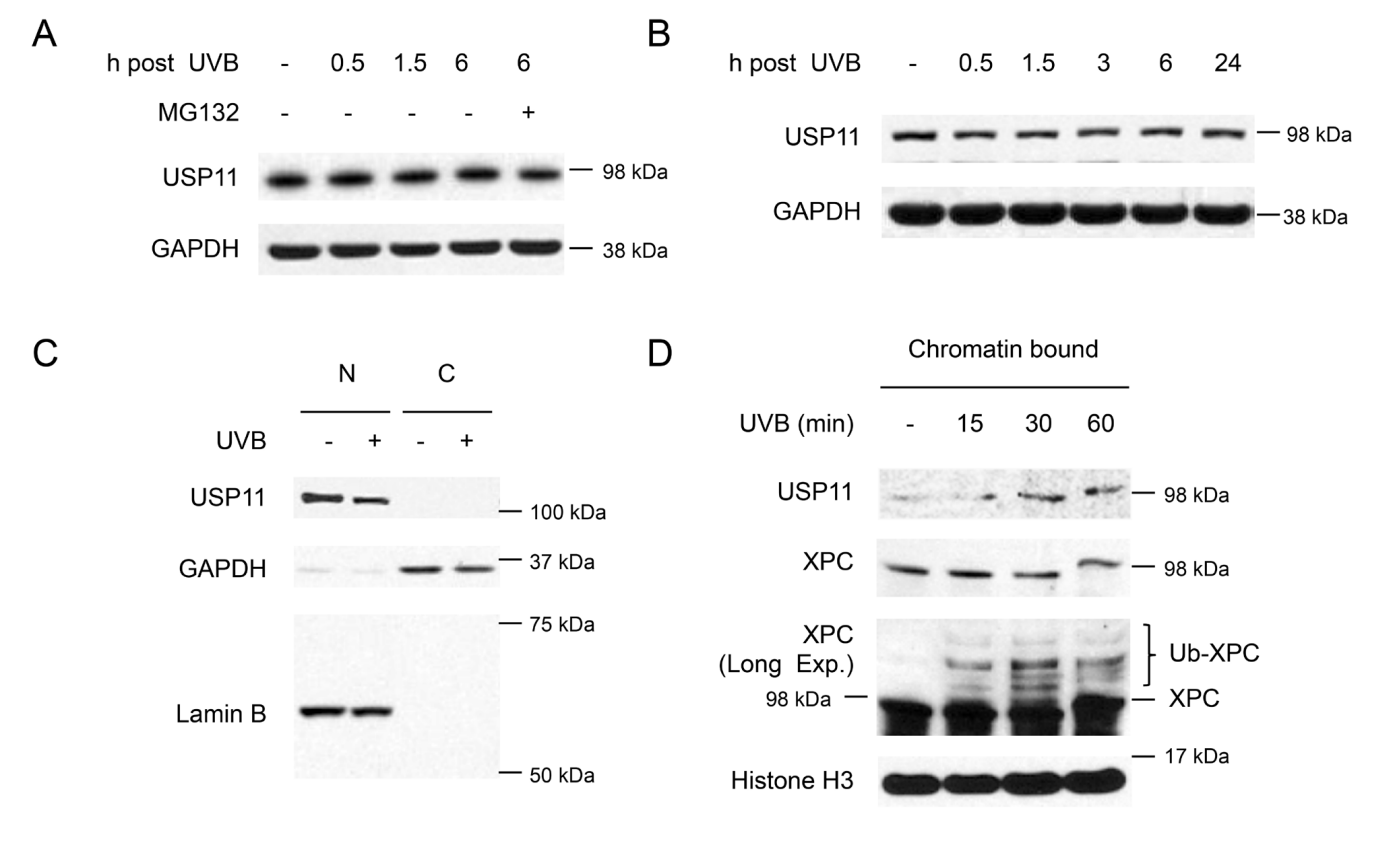
UVB induces USP11 recruitment to the chromatin (**A, B**) Immunoblot analysis of USP11 and GAPDH in HaCaT cells (A), and normal human epidermal keratinocyte (NHEK) cells (B) with or without MG132 treatment (10 µM) 1 h prior to UVB (20 mJ/cm^2^). (**C**) Immunoblot analysis of USP11, GAPDH, and Lamin B using nuclear [N] and cytoplasmic [C] fractions from HaCaT cells with or without UVB (20 mJ/cm^2^, 30 min). (**D**) Immunoblot analysis of USP11, Histone 3, and XPC using chromatin bound protein fractions from HaCaT cells over a time course post-UVB (20 mJ/cm^2^).

To determine the regulation of USP11 by UV irradiation, we first examined the effect of UV on USP11 protein levels and stability. In HaCaT cells, USP11 levels did not change after UV exposure, nor did they increase with MG132 treatment, indicating that UV does not regulate USP11 levels or stability (Figure 5A). Similarly, USP11 levels did not change after UV exposure in NHEK cells, confirming that UV does not regulate USP11 protein levels (Figure 5B). Another mechanism by which UV could regulate USP11 is by affecting its localization. USP11 is mainly localized in the nucleus, and UVB irradiation had no effect on USP11 localization in the nucleus (Figure 5C). However, UV exposure increased USP11 protein levels in the chromatin-bound protein fraction, indicating that UV irradiation induces USP11 recruitment to the chromatin in parallel with XPC ubiquitination in response to UV-induced DNA damage (Figure 5D).

### UVB induces physical interaction of USP11 with XPC dependent on XPC ubiquitination levels

**Figure 6:**
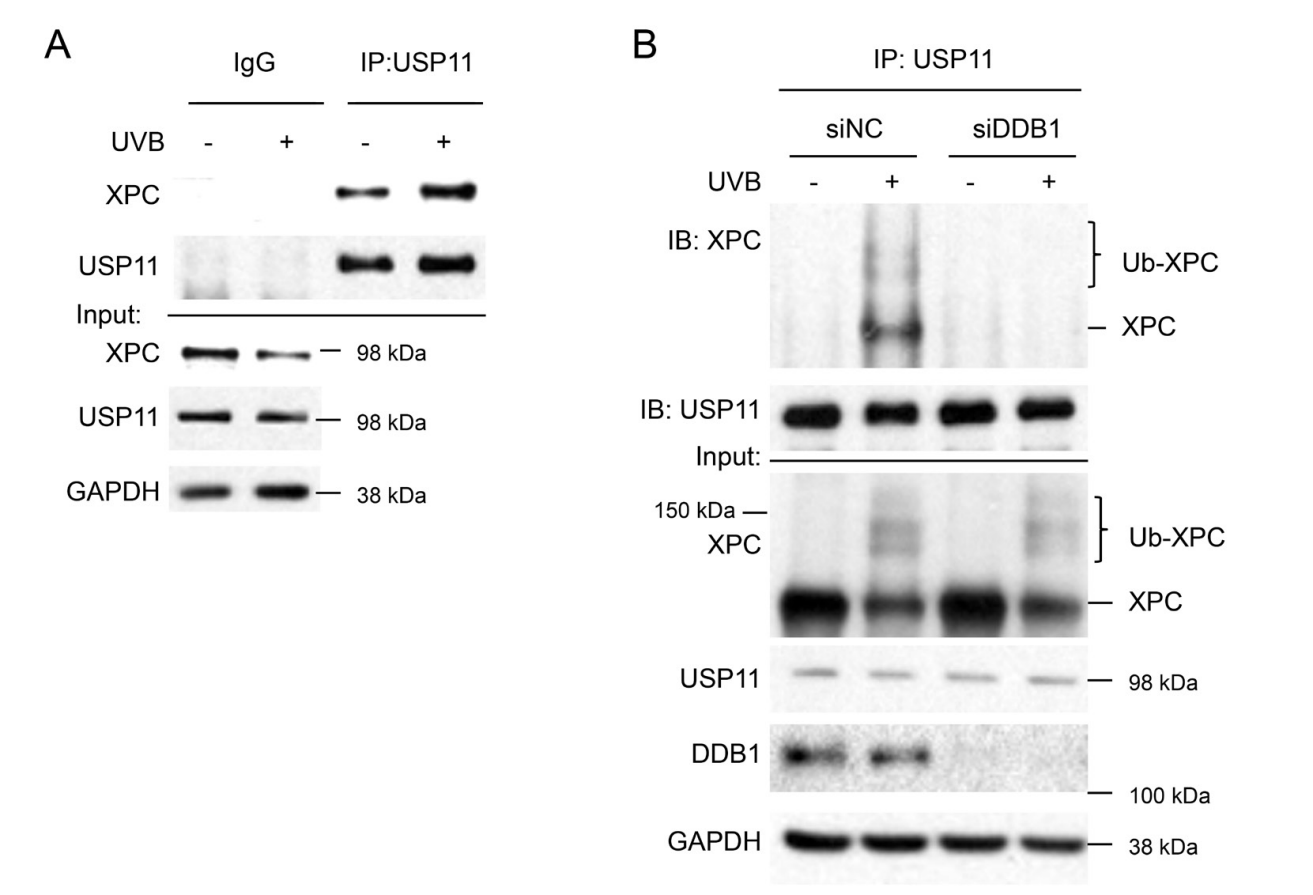
UVB induces USP11 interaction with XPC dependent on XPC ubiquitination levels (**A**) Immunoblot analysis of XPC, USP11, and GAPDH following immunoprecipitation using control species matched IgG and anti-USP11 antibody in HaCaT cells treated with or without UVB (20 mJ/cm^2^, 1.5 h). (**B**) Immunoblot analysis of XPC, USP11, DDB1, and GAPDH in total cell lysates (input) or following immunoprecipitation using anti-USP11 antibody in HaCaT cells transfected with siRNA targeting DDB1 (siDDB1) or non-targeting control siRNA (siNC), and then treated with or without UVB (20 mJ/cm^2^, 1 h).

To determine whether UV regulates USP11 interaction with XPC, we performed co-IP for USP11 and immunoblotted for XPC. We found that USP11 and XPC indeed interacted, and that UV irradiation increased USP11-XPC interaction (Figure 6A). To determine whether UV-induced XPC ubiquitination regulates USP11 interaction with XPC, we determined the effect of DDB1 knockdown on USP11-XPC interaction, since DDB1 is a critical protein in the UV-DDB complex that mediates UV-induced XPC ubiquitination [43]. DDB1 knockdown reduced the UV-induced XPC ubiquitination levels and inhibited USP11-XPC interaction as compared with control cells (Figure 6B). These results demonstrate that XPC ubiquitination levels are critical for UV-induced interaction of USP11 with XPC. We also found that USP11 did not interact with other NER factors or chromatin factors (data not shown), suggesting that USP11 acts *via* its interaction with XPC in the NER process.

### USP11 is down-regulated in mouse skin with chronic UV exposure, and in human and mouse skin tumors

**Figure 7:**
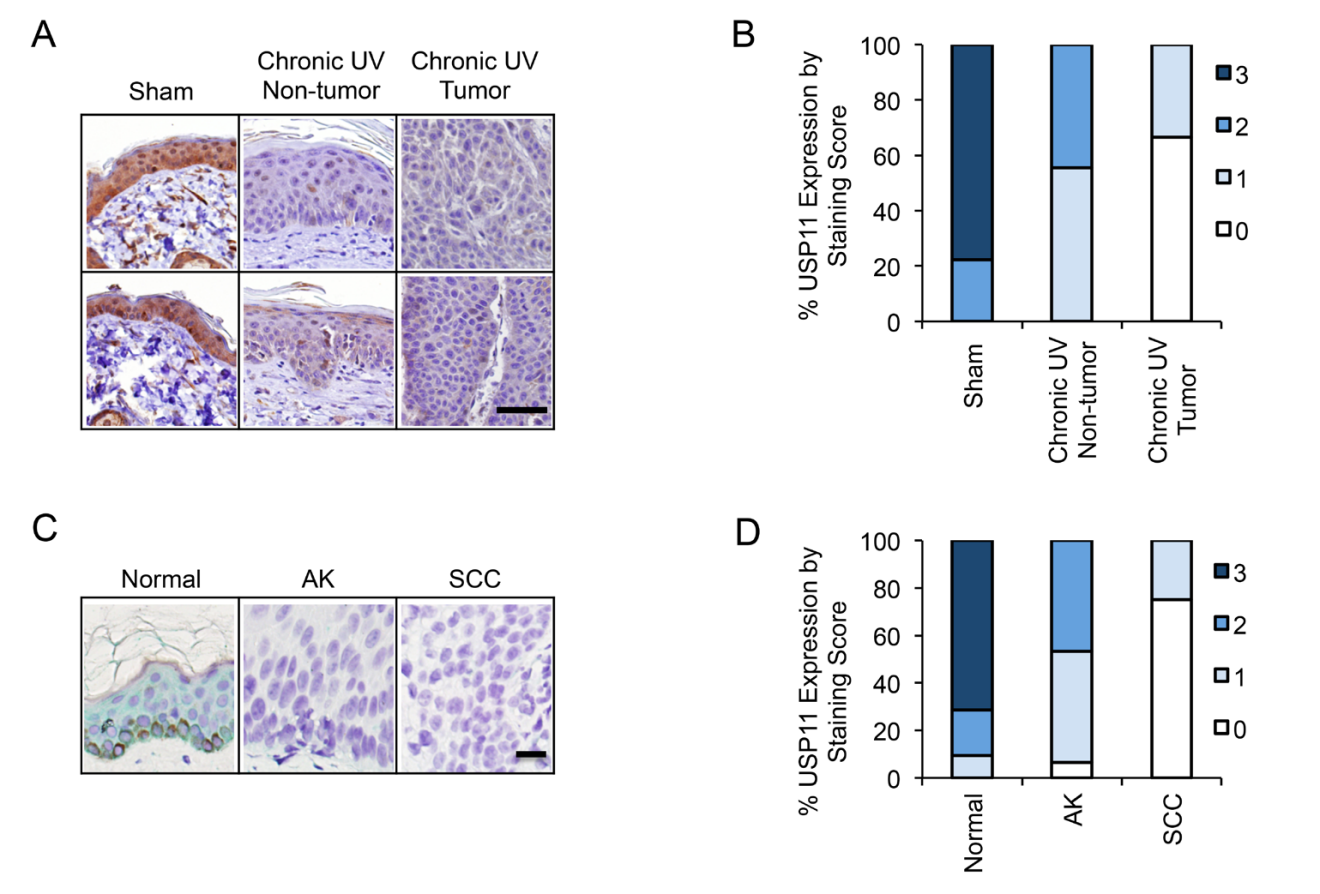
USP11 is down-regulated in mouse skin with chronic UV exposure, and in human and mouse skin tumors (**A**) Representative immunohistochemical analysis of USP11 protein levels (brown) in sham or chronic UVB-irradiated mouse skin with or without tumor. Scale bar, 50 μm. (**B**) Percentage of samples (in stacked column format) for each score of USP11 protein levels in chronic UVB-irradiated mouse skin (tumor and non-tumor) and sham-treated mouse skin (*n* = 9). (**C**) Representative immunohistochemical analysis of USP11 protein levels (green) in normal human skin (Normal), actinic keratosis (AK), and squamous cell carcinoma (SCC). Scale bar, 20 μm. (**D**) Percentage of samples (in stacked column format) for each score of USP11 protein levels in Normal (*n* = 21), AK (*n* = 15) and SCC (*n* = 16) human skin. The Mann–Whitney *U* test was used for statistical analysis (B, D).

To determine the regulation of USP11 by UV exposure and in UV-induced skin cancer, we evaluated the protein levels of USP11 by immunohistochemical staining in skin tissue from sham-irradiated and chronic UVB-irradiated mice (*n* = 9). We found that USP11 levels were high (score 2 or 3) in all sham-irradiated skin tissue (9/9), in ∼45% of the chronic UV-irradiated non-tumor tissue (4/9), and in none of the chronic UV-irradiated tumor tissue (0/9) (Figure 7A and 7B). The differences in USP11 levels among these tissues were found to be statistically significant by the Mann-Whitney *U* test (*p* = 0.0006 for sham versus chronic UV non-tumor tissue, *p* < 0.0001 for sham versus chronic UV tumor tissue, and *p* = 0.0023 for chronic UV tumor versus chronic UV non-tumor tissue). These results indicate that USP11 is down-regulated in UV-irradiated skin and skin tumors, and implicate USP11 as a tumor suppressor in skin cancer.

To determine the role of USP11 in human skin cancer, we evaluated the protein levels of USP11 by immunohistochemical staining in normal human skin tissue (Normal, *n* = 21), actinic keratosis (AK, pre-malignant, *n* = 15), and squamous cell carcinoma (SCC, malignant, *n* = 16). We found that USP11 levels were high (score 2 or 3) in ∼90% of normal skin tissue (19/21), in ∼46% of AK samples (7/15), and in none of the SCC tissues (0/16) (Figure 7C and 7D). The differences in USP11 levels among these tissues were found to be statistically significant by the Mann-Whitney *U* test (*p* < 0.0001 for Normal versus AK, *p* < 0.0001 for Normal versus SCC, and *p* < 0.0001 for SCC versus AK). These results indicate that USP11 is down-regulated in both AK and SCC as compared with normal skin, and suggest that USP11 acts as a tumor suppressor and that USP11 down-regulation is an early event in human skin cancer development.

## DISCUSSION

**Figure 8:**
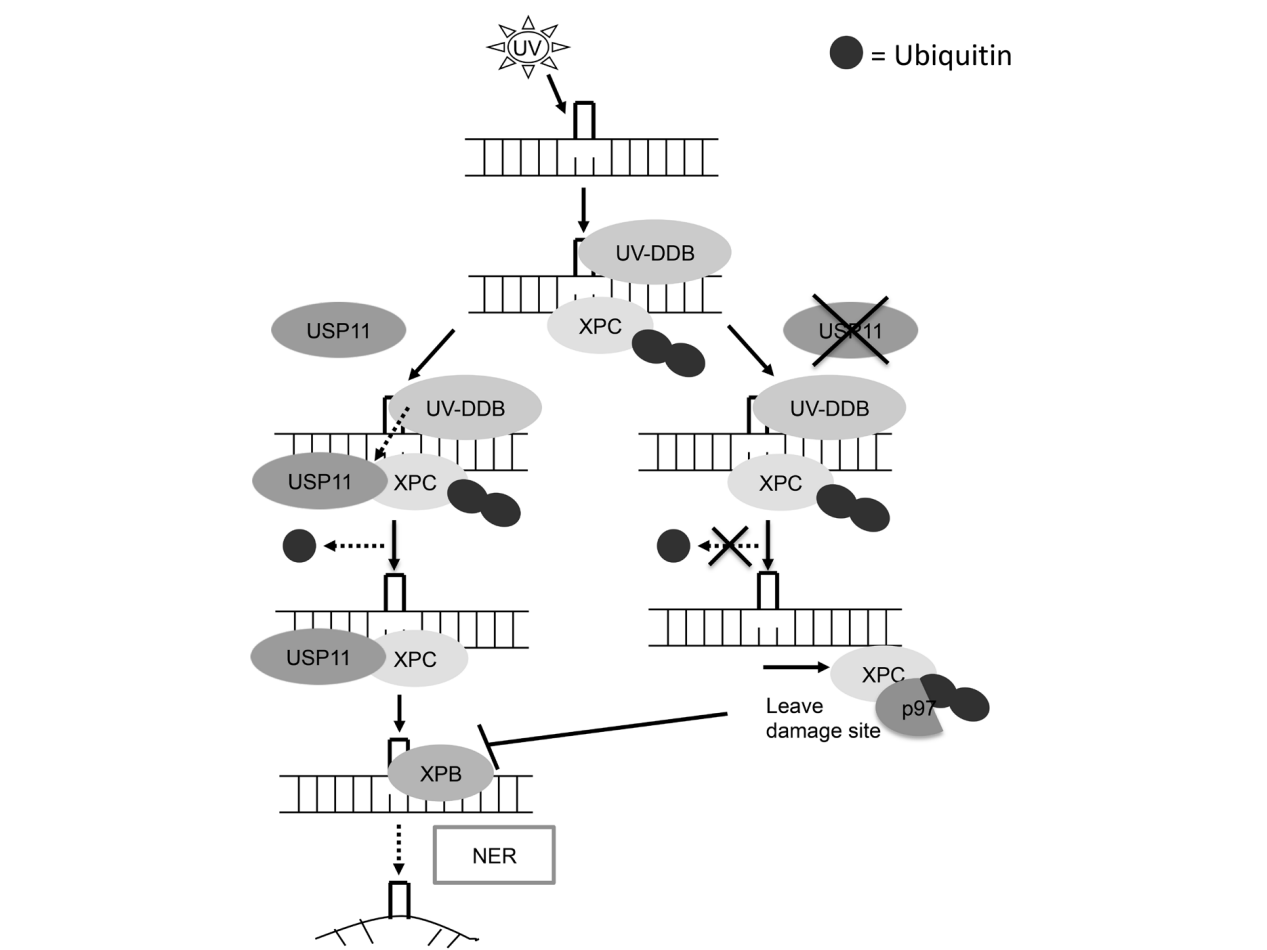
Schematic diagram of USP11 mediated regulation of XPC deubiquitination in nucleotide excision repair UVB induces USP11 recruitment to the chromatin and promotes the interaction of USP11 with ubiquitinated XPC. Then USP11 deubiquitinates XPC and promotes the proper association of XPC with the DNA damage site for positively regulating nucleotide excision repair.

USP11 functions in various pathways and biological processes including TGFβ signaling, pro-inflammatory signaling, viral replication, and NF-κB signaling as well as DNA double-strand break repair [30-38]. However the role of USP11 in UV-induced DNA damage repair is unknown. In this study, we have identified a novel function of USP11 in UV damage repair. We found that USP11 positively regulates the NER process (Figure 8). At the molecular level, USP11 regulates deubiquitination of XPC and its retention at the DNA damage site following UV damage. Since CPD, and not 6-4PP, is responsible for UV-induced skin carcinogenesis, the positive regulation of CPD repair by USP11 suggests a tumor suppressor role of USP11 in skin cancer [40]. Furthermore, USP11 is down-regulated in mouse skin with chronic UVB irradiation and skin tumors from mice and humans. Our findings demonstrate a crucial role of USP11 in UV-induced DNA damage repair and suggest USP11 as a tumor suppressor in skin cancer.

Ubiquitination of XPC has a significant impact on XPC’s function in NER [20-23]. Recently USP7 has been identified as a deubiquitinase for XPC, preventing XPC degradation and promoting the NER process [28]. Here we identify another deubiquitinase, USP11, which can deubiquitinate XPC at the chromatin after UV damage. Furthermore, we show that the catalytic activity of USP11 is essential to regulating XPC deubiquitination in the NER process. Since inhibition of USP11 and USP7 individually have been found to regulate XPC deubiquitination, USP7 and USP11 are non-redundant for regulating XPC deubiquitination. However, as USP7 and USP11 have been found to interact with the same Polycomb complex components [44], it is possible that USP7 and USP11 may interact to regulate NER. It is unclear yet whether they might act synergistically to regulate XPC deubiquitination in NER. Further studies are needed to elucidate whether USP7 and USP11 act synergistically, or which deubiquitinase plays a dominant role, to regulate XPC deubiquitination in NER. USP11 was found to preferentially act on K63 ubiquitin chain linkages [45]: it would be interesting to elucidate the ubiquitin linkage type and the upstream ubiquitin ligase for USP11-mediated deubiquitination of XPC, as well as the amino acid sites on XPC being deubiquitinated by USP11.

Our results also demonstrated that XPC ubiquitination levels regulate UVB-induced USP11-XPC interaction. Reduction in XPC-ubiquitination levels by DDB1 knockdown abolished UVB-induced USP11-XPC interaction. This is further supported by the association of USP11’s recruitment to the chromatin with the parallel XPC ubiquitination. We also found that USP11 did not interact with other NER factors and chromatin factors that could affect NER capacity after UV exposure, suggesting that XPC is the downstream effector of USP11 in the NER process. Although a recent study suggested that USP11 levels may decrease post-UVC insult (50 mJ/cm^2^) [37], we did not find any change in USP11 levels after UVB damage (20 mJ/cm^2^). This might be due to the difference in the cellular model systems, U2OS cells [37] versus keratinocytes in our study, or the different types or dose of UV radiation used. Future investigation will elucidate the specific response to UV radiation in different cell types.

Furthermore, we found that USP11 promotes the damage recognition function of XPC in NER, by preventing premature dissociation of XPC from the DNA damage site. In addition to efficient XPC recruitment to the DNA damage site [46], proper XPC retention at the damage site is critical for efficient NER. Conversely, when XPC dissociation is delayed beyond the optimum time, it hinders access and recruitment of the downstream NER factors to the damage site and decreases NER capacity, underscoring the importance of the appropriate duration for XPC retention at the damage site [26, 41]. In this study, we also found that USP11 inhibition decreases XPB recruitment to the DNA damage site. Our results suggest that USP11 inhibition mediated premature dissociation of XPC from the damage site compromises XPC function to recruit downstream NER factors like XPB to the DNA damage site. We found that USP11 prevents premature dissociation of XPC from the damage site through inhibiting XPC removal by the ubiquitin-selective segregase VCP/p97. Since VCP/p97 mediates removal of ubiquitinated XPC from the DNA damage site to impact genomic stability [41], it is likely that deubiquitination of XPC by USP11 prevents VCP/p97 interaction with ubiquitinated XPC and subsequent removal of XPC from the damage site.

The role of USP11 in cancers is complex. USP11 acts as a tumor suppressor in lung adenocarcinoma and brain tumors [47, 48], but has tumor promoting characteristics in colon cancer, melanoma, pancreatic cancer, and cervical cancer [49-53]. However, the significance of USP11 in skin cancer is unknown. We found that human and mouse skin tumors associated with UV damage show down-regulation of USP11, suggesting that USP11 acts as a tumor suppressor in skin cancer. Additionally, pre-cancerous AK in human skin and UVB-irradiated non-tumor mouse skin showed a decrease in USP11 protein levels in the epidermis as compared with normal skin. Our findings suggest that USP11 functions as a tumor suppressor in the early stages of skin carcinogenesis associated with UV exposure. The function of USP11 in promoting the NER process further supports the tumor suppressive role of USP11 in skin cancer. It remains unknown how chronic UV radiation down-regulates USP11. It is possible that chronic UV exposure alters the microenvironment of the skin, which can lead to USP11 down-regulation. It is also possible that UV exposure causes inactivating mutations in USP11, leading to down-regulation of USP11 or its activity. Future investigation will be needed to elucidate how chronic UV down-regulates USP11 levels. Such insights into mechanisms of USP11 down-regulation by UV exposure and those of promoting USP11activity could lead to translational strategies for prevention of skin carcinogenesis. Moreover, previous studies have indicated that NER could contribute to therapeutic resistance in cancer, especially with agents like cisplatin [54]. Consequently, USP11 inhibitors like mitoxantrone, and more specific USP11 inhibitors developed in the future, have the potential for cancer therapy in skin cancer and other cancers with NER involvement [52, 55].

In summary, we have identified USP11 as a novel post-translational regulator of the NER pathway. Upon UVB exposure, USP11 is recruited to the chromatin and binds to the ubiquitinated XPC. USP11 mediates XPC deubiquitination, thus preventing its premature removal from the damage site by VCP/p97, and promoting proper retention of XPC for its efficient damage recognition function in NER. USP11 is down-regulated in mouse skin with chronic UVB irradiation and skin tumors from mice and humans. Our data indicate that USP11 is a positive regulator for NER, and suggest that USP11 acts as a tumor suppressor in UV-induced skin cancer. These insights into the mechanism of USP11 action on XPC deubiquitination and NER suggest that USP11 could be a promising target for treatment of skin cancer. Moreover, the mechanisms delineated here are also relevant to other NER-associated cancers, such as lung and brain cancers [6, 15, 17].

## MATERIALS AND METHODS

### Human skin tumor samples

All human specimens were studied after approval by the University of Chicago Institutional Review Board. Formalin-fixed, paraffin-embedded tissue blocks were obtained from the archives in the tissue bank of Section of Dermatology, Department of Medicine, University of Chicago. Non–sun-exposed nonlesional normal epidermis, AK, and SCC were used for immunohistochemical analysis.

### Animal treatments

All procedures were approved by the University of Chicago Institutional Animal Care and Use Committee. Female hairless SKH-1 mice (4-6 weeks old, Charles River Laboratories) were randomized and exposed to UVB (100 mJ/cm^2^, dose without visible sunburn) dorsally or sham-irradiated, three times a week for 25 weeks. Mouse skin or tumors were fixed in formalin and used for immunohistochemical analysis.

### Cell culture

Human HaCaT keratinocytes (kindly provided by Prof. N. Fusenig), human embryonic kidney cells HEK293T (ATCC), and HEK293T USP11-knockout cells (ΔUSP11, kindly provided by Dr. Daniel Durocher) were cultured in a monolayer in 95% air/5% CO_2_ (vol/vol) at 37°C in Dulbecco’s modified Eagle’s medium (DMEM) supplemented with 10% FBS, 100 units/mL penicillin, and 100 μg/mL streptomycin (Invitrogen). Normal human epidermal keratinocytes (NHEK) cells (Clonetics, Lonza) were cultured in KGM Gold BulletKit medium (Clonetics, Lonza) according to the manufacturer’s protocol. Cells were tested for mycoplasma and were not recently authenticated by STR profiling.

### UVB radiation

Cells were irradiated with UVB using UV Stratalinker 2400 with UVB bulbs (Stratagene) after washing twice with PBS as described previously [56-59]. Control samples were treated similarly and sham irradiated. The Goldilux UV meter with a UVB detector (Oriel Instruments) was used to monitor the UVB dose weekly. There is no UVC emission from our system.

### siRNA and plasmid transfection

siRNA targeting human USP11 or DDB1 (*ON-TARGETplus SMARTpool*) and Control siRNA (*ON-TARGETplus Non-targeting siRNA)* were purchased from GE Healthcare Dharmacon Inc. Nucleofector (Amaxa, Gaithersburg, MD) was used to electroporate cells with siRNA as previously described [57, 60, 61]. pRK5myc plasmids with wild-type (WT) and C275/283S mutant (csmt) USP11 were kindly provided by Dr. Ruey-Hwa Chen. The plasmids were transfected into HEK293T ΔUSP11 cells using X-tremeGENE 9 according to the manufacturer’s instructions (Roche) as described previously [62].

### Lentiviral production and infection

Human shUSP11 (USP11 MISSION shRNA TRCN0000011090, Sigma-Aldrich) and shCon (obtained from Seungmin Hwang) constructs were co-transfected with pCMVdelta8.2 and pVSV-G plasmids into 293T cells to produce lentiviral particles [62]. The lentivirus was used to infect HaCaT cells and stable cell lines were selected using puromycin.

### Western blotting

Western Blotting was performed as described previously using an SDS-PAGE electrophoresis system [63]. Briefly, cells were lysed in RIPA buffer (Pierce, Rockford, CA) supplemented with Protease and Phosphatase inhibitor cocktail (Thermo Scientific) and harvested. Equal amounts of protein were subjected to SDS-PAGE electrophoresis and electrophoretic transfer to nitrocellulose membranes. 5% nonfat milk in TBST (Thermo Fisher Scientific) was used to block membranes prior to probing with primary and secondary antibodies. The following antibodies were used: XPC (Sigma-Aldrich), USP11 (Bethyl Laboratories, Inc.), GAPDH, Histone H3, DDB1 (Santa Cruz), and myc (Cell Signaling Technology).

### Determination of CPD damage in genomic DNA by immuno-slot-blot assay

Determination of CPD using a slot blot assay was performed as previously described [58, 59, 64]. Briefly, DNA was extracted from cells collected at various times after UV exposure using the QIAamp DNA Mini Kit (Qiagen, Valencia, CA). The absorbance at 260 nm from a NanoDrop 1000 (NanoDrop products, Wilmington, DE) was used to determine the concentration of DNA. CPD monoantibody (TDM-2, Cosmo Bio Co., Koto-Ku, Tokyo, Japan) was used to quantify CPD in the DNA with slot blot (Bio-Rad). To determine repair kinetics, percentage (%) repair was calculated by measuring optical density at the specified times and comparing it to that at time zero hours, since at zero hours 100% of the CPD damage was present after UVB prior to repair.

### Chromatin fractionation

The chromatin-bound protein fraction was extracted from cells using the Subcellular Protein Fractionation Kit for Cultured Cells (Thermo Fisher Scientific #78840) according to the manufacturer’s instructions. The resulting chromatin-bound protein fraction was analyzed by Western blotting.

### Local UV irradiation and fluorescent labeling

The local UV irradiation procedure was carried out as previously described, with some modifications [11, 65]. Briefly, cells were UVC-irradiated (254 nm, 100 J/m^2^) through an isopore polycarbonate filter with 5-μm diameter pores (Millipore Co., Bedford, MA). After incubation for indicated times, cells were fixed, permeabilized, and DNA was denatured with 2M HCl for 30 min at room temperature. Blocking was performed using 5% normal goat serum in PBS (Invitrogen, Carlsbad, California) for 30 min at room temperature, followed by incubation with primary and secondary antibodies for 30 min at 37°C. The samples were mounted in Prolong Gold Antifade Reagent with DAPI (Invitrogen, Carlsbad, California). The antibodies used were CPD (TDM-2, COSMO BIO Co.), XPC (Santa Cruz, sc30156), XPB (Santa Cruz, sc-293), Alexa Fluor 488 F(ab’)_2_ fragment goat anti-mouse IgG and Alexa Fluor 568 goat anti-rabbit IgG antibodies.

### Immunoprecipitation

Immunoprecipitation was carried out as described previously using anti-USP11 (Bethyl Laboratories, Inc., Rabbit, A301-613A) antibody [62].

### Immunohistochemical analysis

Immunohistochemical analysis for USP11 levels was carried out by using anti-USP11 antibody (Atlas Antibodies, Cat # HPA037536) by the Immunohistochemistry core facility at the University of Chicago. The protein levels were visualized with the diaminobenzidine (DAB) method (brown color) in mouse skin, and Vina Green™ Chromogen Kit (green color, to exclude the contribution of endogenous brown pigmentation) in human skin, respectively. USP11 levels in tissue sections were scored blindly by two independent investigators as strong (3), moderate (2), weak (1), or absent (0), as previously published [66, 67].

### Statistical analyses

Statistical analysis was performed with Prism 5 (GraphPad software, San Diego, CA, USA). Sample size calculations were performed with StatMate 2.00 (GraphPad). The number of mouse or human tissues used in the *in vivo* models is determined based on 80% power, a two-sided test with a significance level of 0.05. Two independent investigators double blindly scored the normal and tumor sections as no staining (0), weak (1), moderate (2) or strong (3) for USP11. Data were shown as the mean of three independent experiments and analyzed by Student’s *t-*test (two-tailed). Immunohistochemical analysis was analyzed by the Mann-Whitney *U* test (two-tailed). *p*-value <0.05 was considered statistically significant. Error bars were shown as standard errors of the mean (S.E.).
